# COVID 19 pneumonia in Down syndrome patients: About 2 cases

**DOI:** 10.1016/j.amsu.2021.102324

**Published:** 2021-04-22

**Authors:** Abderrahim El Kaouini, Abdelilah El Rhalete, Mohammed Aabdi, Mohammed Azizi, Mohammed Maarad, Choukri Bahouh, Houssam Bkiyer, Brahim Housni

**Affiliations:** Intensive Care Unit and and Anesthesiology Department, Mohammed VI University Hospital, Oujda, Morocco

**Keywords:** COVID-19 pneumonia, Down syndrome, Pro-inflammatory factors, Immune deregulation, Cytokinic storm

## Abstract

**Introduction:**

Covid-19 is a severe emerging infection with high rate of mortality. Patients with Covid-19 and Down syndrome represent a high rate of morbidity and mortality.

**Case presentation:**

Case 1: A 27-year-old white male with Down's syndrome admitted to the ICU for Covid-19 infection with lung damage of 30–50%. The patient improved and referred to the pulmonology department.

Case 2: A 49-year-old man admitted to the ICU for Covid-19 infection with a lung damage of 50%. The evolution was lethal and he passed away after 12 days of his admission.

**Conclusion:**

People suffering from Down syndrome should be given priority in the management of acute respiratory distress following infection with SARS COV2, or even candidates for early immunosuppressive treatment and possible vaccination once started.

## Highlights

• SARSCoV-2 is an emerging pandemic with high rate of morbidity and mortality.
• In this paper we will report the case of two patients with SARS-CoV-2 in patients with Down syndrome.
• Following the presence of a high frequency of respiratory diseases with a high rate of co-morbidities associated with Down syndrome, all these factors can lead to severe clinical presentations of COVID19 in this type of patient.

## 1 Introduction

SARS-CoV-2 is the agent responsible for the global pandemic of Corona virus 19 (COVID-19). Clinically, this disease can either be asymptomatic or severe and fatal. The severity of COVID 19 is mainly due to pro-inflammatory factors which generate an increased inflammatory and immune response resulting in acute respiratory distress syndrome associated with organ failure and death [1]. Down's syndrome is an inherited disease, the most common in humans, secondary to the presence of an extra copy of chromosome 21, this syndrome is associated with immune dysfunction with a predisposition to autoimmune diseases and an anatomical differences in the upper respiratory tract predisposing to a high frequency of respiratory diseases mainly lower respiratory infections [2].

## 2 Presentation of the cases

In this work, we report two cases of SARS-CoV 2 in patients with trisomy 21, with a polymerase chain reaction (PCR test) on a nasopharyngeal swab on admission to the intensive care unit. The swab was positive for SARS-CoV 2 and a thoracic tomodensitometry with injection (CT scan) was positive for frosted glass pneumonia on both sides.

### 2.1 Case 1

A 27 year old white male with Down's Syndrome, with a history of hypothyroidism on l-thyroxine, was admitted to the intensive care unit on 5 November 2020 from the COVID 19 emergency department due to a dyspnea that had been progressively worsening over the last six days associated with an unspecified fever, myalgia and generalised asthenia. The clinical examination at the patient's admission was objective: Neurological stability with a Glasgow score 15/15, body temperature 37.6 °C, normocardia with a heart rate at 76 bpm, blood pressure (BP) 135/65 mmHg, polypneic at 32 cycles per minute with a peripheral oxygen saturation (SpO 2) at ambient air (AA) 75% with a PH level at 7.38, the arterial oxygen pressure level (PaO2) at 51 mmhg in ambient air and the arterial CO2 level (PaCO2) at 39 mmhg, the PaO 2/FiO 2 (P/F) ratio of 242 mmHg. The nasopharyngeal swab PCR test was positive for SARS-CoV-2 and a thoracic tomodensitometry with injection (CT scan) showed a bilateral ground glass pneumonia with an estimated 30–50% parenchymal involvement and no sign of pulmonary embolism, biologically the patient had lymphopenia 650 cells/μl, high levels of C-reactive protein (CRP) (130 mg/L, normal range <10), procalcitonin at 3 μg per litre (normal range < 0.5), D-dimers (410 ng/ml) were slightly above the normal value (<250 ng/ml), an increase in LDH (310 U/L), and ferritin (1870 ng/ml), normal liver and kidney function, and an ionogram returning without particularities. The treatment was oxygen supplementation of 10 L per minute in a high concentration mask, an antibiotic therapy based on piperacillin-tazobactam was started, corticotherapy with dexamethasone 6 mg per day and preventive anticoagulation with enoxaparin subcutaneously (4000 IU/day). The evolution has been good with a P/F ratio on day 7 of hospitalisation constantly higher than 280–300 mmHg with a significant reduction in respiratory work and oxygen supplementation from 10 L per minute to 4 L per minute under glasses. Biologically, there was an increase in the lymphoct rate from 650 to 100 cells/μl and a normalisation of CRP and procalcitonin levels. The patient was transferred to the pneumology department on 13 November 2020 for further treatment.

### 2.2 Case 2

A 49 year old man with Down's syndrome, with a history of well controlled epilepsy on Tegretol, congenital hydrocephalus, was admitted to the intensive care unit from the COVID 19 emergency department on December 1, 2020 due to 9 days of dyspnea associated with myalgia and anosmia. The physical examination at the admission of the patient was objective: a Glasgow score 15/15, body temperature 36.8 °C, a heart rate at 89 bpm, blood pressure (BP) 145/70 mmHg, polypneic at 35 cycles per minute with a peripheral oxygen saturation (SpO 2) at ambient air (AA) varies between 68 and 70% with a PH level of 7, 34, the arterial oxygen pressure (PaO2) at 46 mmhg in ambient air and the arterial CO_2_ (PaCO_2_) at 41 mmhg, the PaO 2/FiO 2 (P/F) ratio of 219 mmHg at the patient's admission. The PCR test on a nasopharyngeal swab was positive for SARS-CoV-2 and a thoracic tomodensitometry with injection (CT scan) showed images suggestive of SARS-CoV-2 pneumonia with an estimated 50% parenchymatous degree of damage and no sign of pulmonary embolism. The patient had deep lymphopenia of 350 cells/μl, The results are: high levels of C-reactive protein (CRP) (210 mg/L, normal range < 10), positive procalcitonin level at 8 μg per litre (normal range < 0.5), positive D-dimer level (410 ng/ml), normal range <250 ng/ml), an increase in LDH (560 U/L), and ferritin (2010 ng/ml), normal liver and kidney function, and a returning ionogram without peculiarities. The conduct held was oxygen supplementation 10 L per minute in a high concentration mask with the objective of peripheral oxygen saturation 88–92%, an antibiotic therapy based on piperacillin-tazobactam was started, corticotherapy with dexamethasone 6 mg per day and preventive anticoagulation provided by subcutaneous enoxaparin (4000 IU/day), Tergretol 400mg prolonged release per day. The evolution was marked by a progressive reduction of the P/F ratio to 150 mmHg then to 100 mmHg with an increase in oxygen supplementation up to 15 L/min followed by a continuous supplementation in positive airway pressure (CPAP) with an increase in the markers of inflammation. Subsequently and given the non-improvement of his condition, the patient underwent endotracheal intubation, mechanical ventilation with deep sedation and continuous curarisation associated with a session of ventral decubitus of 16 hours. He was ventilated mechanically for 4 days, the evolution was unfavourable, the patient died on December 12, 2020.

## 3 Discussion

We presented in this work two cases of patients with Down syndrome who were affected by COVID 19. Among our two patients, the evolution of one patient was unfavourable. Our two patients had different clinical features of Down syndrome, case 2 presenting with Down syndrome whose features were much more serious and complex, in the literature more than 80 clinical features of Down syndrome with variation in severity have been reported [3], so infectious complications are more frequent and severe in patients with Down syndrome due to the anatomical differences in the upper respiratory tract, the prognosis is much worse in the presence of associated cardiac malformations [4]. the second patient was 49 years old, close to the maximum life expectancy for Down syndrome patients, advanced age is considered to be a predictor of VIDOC-related mortality 19 [1]. The evolution was unfavourable for the second patient with an increase in oxygen supplementation up to 15 L/min followed by continuous positive airway pressure (CPAP) supplementation, this increase in oxygen supplementation was associated with a very significant increase in inflammation markers. In the case of SARS-CoV-2 infections, the worsening of the respiratory state of patients is strongly linked to a highly exacerbated immune response [5] to the virus leading to an increased release of inflammatory cytokines [6]. In the light of these considerations, the different evolution of the two patients may be due to (i) the different clinical features of Down syndrome, (ii) advanced age, (iii) the different immunological response and host-virus interaction, this host-virus interaction resulted in a very important cytokine storm in the second patient.

## 4 Conclusion

Down's syndrome is a hereditary disease, the most frequent in humans, characterised by an anatomical difference in the upper respiratory tract, predisposing to a high frequency of much more severe respiratory diseases in the case of associated malformations, particularly cardiac. People suffering from Down syndrome should be given priority in the management of acute respiratory distress following infection with SARS COV2, or even candidates for early immunosuppressive treatment and possible vaccination once started.

This case report follows care guidelines [7].

## Ethical approval

Not applicable, this is a case report.

## Sources of funding

This research was not funded.

## Author contribution

Dr. Abderrahim El Kaouini: is the principal investigator + corresponding author.

Dr RHLAET ABDELILAH contributor.

Dr AABDI Mohammed contributor.

Dr Mohammed Azizi: contributor.

Dr. Mohammed Maarad: contributor.

Dr BAHOUH choukri contributor.

Prof. Houssam Bkiyer: supervised the research project. *

Prof. Brahim Housni supervised the research project. *

All authors approved the final version of the manuscript.

## Research registration number

Not applicable this is a case report.

## Guarantor

Dr. Abderrahim El Kaouini.

## Consent

The 2 patients fully consented to the study.

## Declaration of competing interest

The authors declare no conflict of interest.
